# Assessing the relationship between armed conflict and infectious disease incidence in Six Sub-Saharan Countries: Implications for Emergency Departments in conflict areas

**DOI:** 10.1016/j.afjem.2025.100911

**Published:** 2025-09-26

**Authors:** Andrew Holzman, Daniel Olinga, Jacob Busingye, Douglas Rappaport

**Affiliations:** a Mayo Clinic Alix School of Medicine, AZ, USA; b Department of Emergency Medicine, Mbarara University of Science and Technology, Uganda; c Department of Emergency Medicine, Mayo Clinic, AZ, USA

**Keywords:** Conflict, Infectious disease, Tetanus, Measles

## Abstract

**Introduction:**

This study aimed to update and expand on prior ecological analyses by examining within-country and cross-national correlations between conflict deaths and infectious disease incidence in six neighboring sub-Saharan African countries.

**Methods:**

We analyzed six countries including Uganda, Rwanda, the Democratic Republic of the Congo, Central African Republic, South Sudan, and Burundi using conflict death data from the Uppsala Conflict Data Program and disease incidence data from the World Health Organization databases. Data were analyzed for the maximum period covered in the UCDP for each country, in general from 1990 to the present. Seven diseases were examined: malaria, tuberculosis, human immunodeficiency virus, tetanus, pertussis, measles, and yellow fever. We assembled matrices of regressions between conflict deaths and disease incidence within and across countries. The approach of this study was hypothesis-generating, with the intent to flag associations worthy of further consideration.

**Results:**

Within-country analyses identified strong positive associations in Burundi (e.g., tuberculosis: R² = 0.81, *p*<0.001; tetanus: R² = 0.59, *p*<0.001) and Uganda (e.g., tuberculosis: R² = 0.56, *p*<0.001; malaria: R² = 0.42, *p*<0.001). Weaker but statistically significant correlations were also observed with measles in Uganda (R² = 0.13, *p* = 0.04) and Burundi (R² = 0.29, *p* = 0.001). Cross-nationally, we observed strong correlations between conflict deaths in Burundi and tuberculosis incidence in Uganda (R² = 0.86, *p*<0.001) and conflict deaths in South Sudan and malaria in the Democratic Republic of the Congo (R² = 0.64, *p*<0.001).

**Conclusion:**

We confirm prior reports of malaria incidence association with armed conflict while introducing potential new associations involving tetanus and measles. Our hypothesis-generating approach supports further research. Emergency physicians and health systems in conflict-affected and adjacent regions should anticipate shifts in disease burden and consider preparedness strategies.

African Relevance

• Emergency Departments in conflict-affected regions in central Africa should be prepared for changes in incidence of infectious disease (in particular, malaria and tuberculosis but also, as uniquely demonstrated in this study, measles and tetanus) during conflict flares.
• Index of suspicion for infectious processes should be adjusted during times of conflict in neighboring regions to account for changes in the incidence both of infectious diseases associated with conflict and also for risk factors unique to refugee populations.
• Immunization takes on critical importance in times of conflict, as displaced persons will come into increased contact with pathogens. Immunization and post-exposure prophylaxis can be important elements of emergency practice during conflict flares.
• Adjustment of cold storage requirements continues to be a priority for the practice of medicine in resource-limited settings, and this study highlights the application of this to tetanus toxoid in conflict-affected regions.

## Introduction

Violent conflict is well established as an important determinant of infectious disease [1]. In the sub-Saharan African context, this has been discussed in the cases of malaria, HIV and tuberculosis (TB). Conflict and the displacement it causes may have impacts on both population exposure to environments representing malaria risks and the efficacy of mosquito control efforts, and this has led to the demonstration in some studies of a quantitative association between conflict and infection [2].

The association of outbreaks of conflict with HIV has also been studied, although the relationship is more complex, with possible rises in infection in the period before maximal conflict mortality [3] This analytical complexity may be due in part to the time-lagged nature of HIV clinical presentation and diagnosis as well as diagnostic challenges specific to conflict-affected regions. HIV incidence may be high among soldiers, who may act as vectors for the disease during war [4].

A positive association with tuberculosis incidence has also been described in some settings [5], although conflict has a complex impact on testing and notification processes [6]. Conflict also presents known challenges to vaccination programs, [7] which compounds the public health challenges posed by refugee movements. One study specifically attributed a measles outbreak in Ethiopia to the problem of vaccine distribution during conflict [8].

Emergency Medicine as a medical specialty has seen substantial growth in recent years in sub-Saharan Africa [9]. Much of the emphasis in Emergency Departments (ED) in conflict zones relates to the management of patients with acute traumatic injuries. Relatively less research has been done to address the rising risk of infectious diseases in times of conflict that are seen in the ED. As an example, response to the recent outbreak of Mpox in the DRC was influenced by conflict in the region, with high case burden among displaced persons and difficulty in disseminating public health messaging, posing a significant hurdle to appropriate response [10].

The impact of armed conflict extends long beyond the acute period when deaths are greatest. Uganda’s situation highlights this well; it houses nearly 2 million refugees, and must cope with flows of both internally and internationally displaced persons [11]. Among its refugee settlements are some of the largest in Africa and the world; for example, Bidibidi refugee settlement is a city in itself with as many as 270,000 homes [12]. The epidemiology of populations in these centers, influenced by the nature of refugee flows and of the conflicts that drive them, is an important consideration for public health in sub-Saharan nations. Instability also persists long after the period of greatest conflict death, and all of the countries discussed here remain subject to the effects of continued, smoldering conflict even after resolution to the major civil wars covered in this paper. While in-depth discussion of this instability is outside our scope here, it continues to be an important driver of health outcomes broadly in the region.

The purpose of this study was to assess the impact of conflict on the incidence of seven diseases in six neighboring sub-Saharan countries– including Uganda, Rwanda, the Democratic Republic of the Congo (DRC), Central African Republic (CAR), South Sudan, and Burundi– both to update the relationships previously described in the literature and to expand the analysis to additional diseases and the threats these pose to ED in these areas. Our study is structured as a correlational matrix evaluation and is therefore hypothesis-generating in nature; positive results of our analysis are susceptible to the risk of spurious correlation and must be examined with scrutiny. Statistically significant relationships will be assessed for the presence of an underlying theory of causation, and where this exists they may be good candidates for further study as falsifiable hypotheses.

## Methods

We conducted an ecological study using publicly available annual data from two primary sources. Conflict fatality counts were obtained from the Uppsala Conflict Data Program (UCDP) geolinked events database [13], and infectious disease incidence data were sourced from the World Health Organization’s Vaccine-Preventable Diseases Monitoring System (WHO VPD) [14] and the Global Health Observatory (WHO GHO) [15].

The study included six neighboring countries in Central and East Africa: Uganda, Rwanda, the Democratic Republic of the Congo (DRC), Central African Republic (CAR), South Sudan, and Burundi. UCDP data was processed by summing the “best estimate” field for conflict deaths for each relevant country across each year for the full coverage of the dataset (1989–2023). Next, data from both the WHO VPD and WHO GHO was matched to years in which conflict data was available to create a panel dataset in which each country’s annual conflict casualty number was linked to the relevant incidences of infectious disease in that country. For each country, where conflict data was null in a given year after the country’s first available conflict data, an entry in the panel dataset was created to ensure overlap of the data for between-country analysis. (This was not possible for South Sudan, where data availability was poor and entries began in 2006). For Uganda, conflict data covered the period from 1989–2023; for Rwanda, 1990–2023, for the DRC, 1989–2023, for the CAR, 1991–2023, and for Burundi, 1990–2023.

We analyzed seven infectious diseases, for which incidence was measured differently between the WHO databases: tetanus, pertussis, measles and yellow fever were measured by incidence per 1 million population (WHO VPD). Malaria was measured by incidence per 1000 population at risk; HIV as infections per 1000 individuals and tuberculosis as incidence per 100,000 (WHO GHO). Diseases were selected for acceptable data availability and to overlap with previous studies of infectious disease in conflict as discussed above.

We constructed matrices of all possible univariate regressions to examine relationships between conflict deaths and disease incidence both within each country and across countries. For each regression, we extracted the coefficient, R-squared (R²), p-value, and number of observations (accounting for years with data availability mismatch).

Data analysis and visualization were performed in R using the dplyr, ggplot2, broom, purrr, stringr, scales, and janitor packages. We applied z-score normalization for time series visualization and used line smoothing to interpolate missing annual data for visualizations only. Given the nature of the datasets used, no ethical approval or waiver was required by our institution for this study.

## Results

**Fig. 1:**
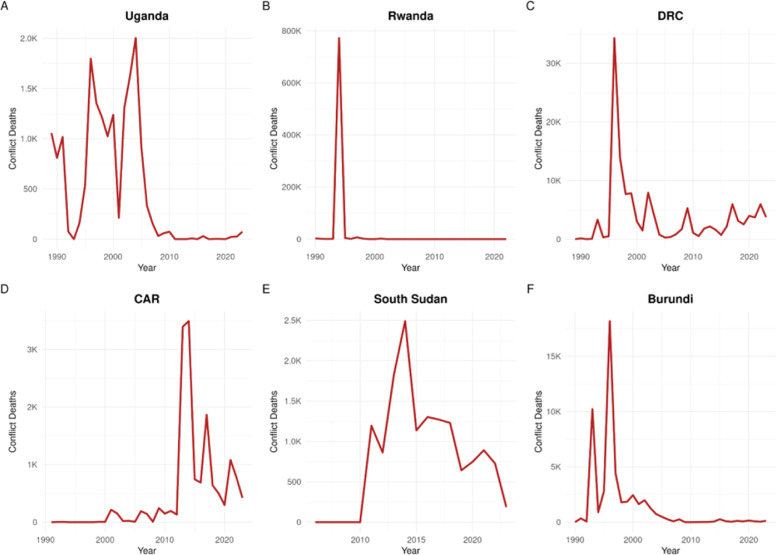
Conflict deaths for each country studied over time.

We began by plotting conflict deaths across time to determine the specific conflicts likely captured by the dataset. The full set of data is shown in Fig. 1.

Table 1 shows the results of statistically significant correlations in the within-country analysis (of a country’s conflict deaths regressed against its disease incidence). N refers to the number of overlapping points available for analysis in the regression. Table 1 shows correlations for all diseases in each country but omits those for which *p*>0.05.

**Table 1:** Statistically significant correlations from within-country analysis of conflict deaths and disease incidence (p < 0.05 only).

Country	Disease	Coefficient	R squared	Correlation	P value	N (years with data overlap)
Uganda	malaria	0.095	0.42	0.652	<0.001	23
Uganda	tuberculosis	0.029	0.56	0.746	<0.001	23
Uganda	HIV	0.001	0.27	0.521	0.002	33
Uganda	measles	0.493	0.13	0.367	0.036	33
Democratic Republic of the Congo	pertussis	−0.007	0.20	−0.445	0.026	25
Central African Republic	HIV	−0.001	0.20	−0.444	0.011	32
South Sudan	measles	−0.238	0.54	−0.735	0.007	12
Burundi	malaria	0.104	0.73	0.857	<0.001	23
Burundi	tuberculosis	0.073	0.82	0.904	<0.001	23
Burundi	tetanus	0.002	0.59	0.771	<0.001	26
Burundi	measles	0.184	0.29	0.536	<0.001	33

**Fig. 2:**
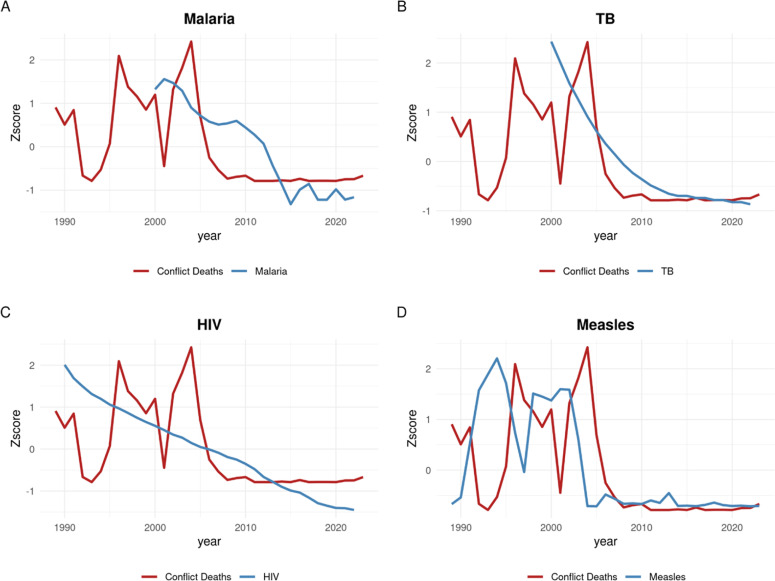
Z-score normalized trends over time for conflict deaths and disease incidences for each disease studied in Uganda.

**Fig. 3:**
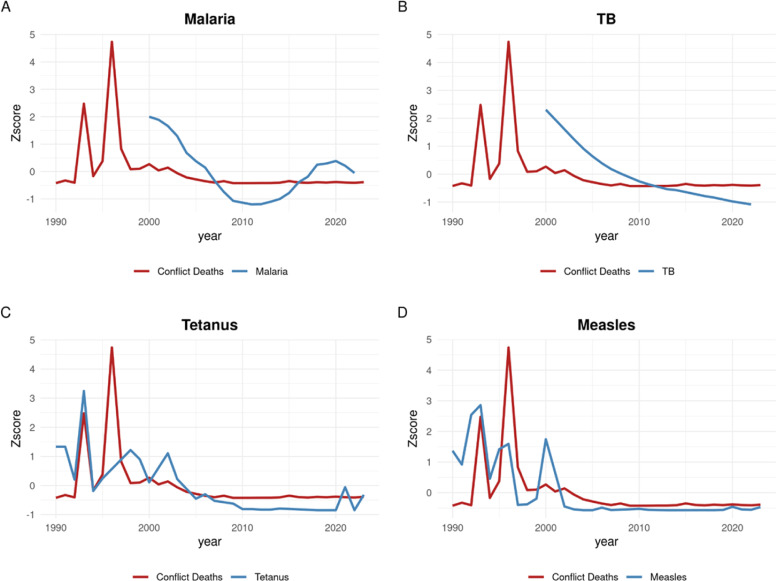
Z-score normalized trends over time for conflict deaths and incidence of each disease studied in Burundi.

Conflict data and disease incidence were plotted across time on a z-score normalized y axis to understand potential trends underlying the correlations reported. The associations found in Uganda and Burundi are shown in Fig. 2, Fig. 3, respectively. Next, a correlational matrix was constructed to compare conflict deaths in each country with disease incidence in each other country. The statistically significant results with R² greater than 0.50 are shown in Table 2. N refers to the number of overlapping values included in the correlation dataset. Table 2 omits correlations for which *p*>0.05 or R^2^ <0.50.

**Table 2:** Correlational matrix results for between-country associations (where p < 0.05 and R squared >0.5).

Predictor	Outcome	Coefficient	R squared	P value	N (years with data overlap)
Conflict deaths (Burundi)	TB (Uganda)	0.031	0.86	<0.001	23
Conflict deaths (Uganda)	Tetanus (South Sudan)	0.127	0.73	0.002	10
Conflict deaths (Uganda)	Measles (South Sudan)	7.100	0.67	0.001	12
Conflict deaths (South Sudan)	Malaria (DRC)	−0.065	0.64	<0.001	17
Conflict deaths(Uganda)	TB (Burundi)	0.069	0.55	<0.001	23

## Discussion

### Conflicts captured by the data

For the countries in question, the UCDP dataset captures a number of complex and interconnected conflicts important to the Great Lakes region of Central and East Africa. Ugandan conflict deaths spiked in the mid 1990s and 2000s, with greatest impact on northern Uganda, with refugee flows primarily involving massive, forced internal displacement [16] but with some reports of movement into South Sudan, especially from conflict in the DRC and CAR north of Uganda [17].

In Rwanda the genocide of 1994v shows a sharp spike in conflict deaths, and contributed to widespread destabilization across the region as refugees and militants left the country, especially to the DRC [18]. The conflict data for the DRC reflects a complex cycle of destabilization and violence within the country since the onset of the First Congo War in 1996 [19]. The data shown for Burundi, reflect civil war from 1993–2005 [20] and refugee flows included displacement to Uganda, Rwanda, the DRC and Tanzania [21]. In the CAR a coup in 2013 led to widespread instability and fighting between several militia factions [22]. South Sudan became independent from Sudan in 2011 after years of fighting with the more developed north, which led to a civil war [23].

### Analysis of observed correlations

Analysis of the within-country correlations yielded several correlations worthy of note. We also note the presence of some negative correlations, which may be due to decreased reporting during times of conflict or due to overfitting of the data related to the small sample size in some cases. Positive correlations were better supported as potentially causal by the existing theoretical evidence and these were pursued for further analysis.

In Uganda, the correlation for measles is low (R² = 0.13, *p* = 0.04) but statistically significant, and the data show multiple peaks in conflict data potentially related to spikes in disease incidence with good overlap between data availability for the two variables. Malaria incidence (R² = 0.42, *p* < 0.001) also shows a convincing temporal association. The correlation with tuberculosis (R² = 0.56, *p* < 0.001) is relatively strong, although the limited availability of disease data makes a meaningful trend difficult to distinguish from one driven primarily by reducing disease incidence over time incidentally following the end of conflict (though this phenomenon could also reflect an underlying causal mechanism). The theoretical basis for suspicion of a causal relationship with infectious disease is high in Uganda, where the nation was thrust into crisis by an extraordinary number of internally-displaced persons living for long periods of time in crowded settings [16]. Many of the internal refugees fled from the less-developed north of the country, possibly explaining increased susceptibility to infectious diseases in this population.

Meaningful correlations were also observed in Burundi. In particular, the data appear to show an especially convincing temporal association between conflict spikes and the incidence of tetanus (R² = 0.59, *p* < 0.001). There is also an apparent correlation to the incidence of measles (R² = 0.29, *p* = 0.001). For TB (R² = 0.81, *p* < 0.001) and malaria (R² =0.73, *p* < 0.001) the correlations observed are strong, and although the primary period of conflict does not overlap with disease data availability, the correlation is still observed in the period following peak conflict deaths.

Between-country data was more challenging. In the case of the Ugandan conflict data’s potential correlation with South Sudanese indicators, the plausibility of refugee flows matching the directionality of the correlation makes the associations worthy of note. The association between South Sudanese conflict data and malaria in the DRC was statistically significant and strong (R² = 0.64, *p* < 0.001). The limited availability of data for South Sudan do present challenges for interpretation of this trend, although underlying the association are known refugee flows matching its direction, especially into the northern DRC [24]. A more complex analysis of this trend could involve investigation of the period before South Sudanese independence and the accompanying civil war, although the limited availability of infectious disease data would continue to present a challenge to such an approach.

### Policy impacts

In summary, our data support previously reported trends between conflict and the incidence of malaria and TB. Our analysis demonstrates how large flows of refugees, often housed in close-quarters, underlie this trend. Less discussed has been the association with tetanus and measles, demonstrated most convincingly in Burundi. This important association has key implications for emergency medicine and EDs across the region.

Of note, access to emergency physicians is limited for much of the population in the area studied. Efforts to inform care therefore should involve interdisciplinary outreach to the variety of primary care and healthcare extension services in the area. In Uganda, for example, the very high population of refugees is served in part by Village Health Teams of four community workers each at the community level. In the Bidibidi refugee settlement, these serve about 30 households each and provide basic health education and management [25]. Training in these extension systems can be limited, but our research could support building programs to target enhanced recognition of communicable diseases tailored to refugee populations entering an area.

The association of conflict with tetanus incidence, perhaps related to refugee movement through densely forested areas or mechanisms of violent injury, has specific implications for emergency practice. Tetanus toxoid vaccine requires cold storage, typically involving a distribution system with a temperature chain keeping the vaccine between 2 and 8 degrees Celsius. This presents challenges in each of the countries analyzed here, where infrastructure is limited and subject to instability during conflict. For this reason, the WHO adopted a roadmap to permit more flexible administration of cold-storage vaccines proven to be safe for storage above 40 degrees Celsius for at least 3 days [26]. In the case of tetanus toxoid, for example, some data suggests it may remain non-inferior at up to 40 degrees Celsius for less than 30 days [27]. Unfortunately, high-quality research has remained sparse and the lack of interest from manufacturers and policymakers on the issue has challenged the rollout of the WHO framework in resource-limited regions [28]. Developing this research could widen the availability of the vaccine to the community and primary healthcare center services in conflict areas discussed above.

Emergency physicians must consider a complex matrix of factors including population disease prevalence and vaccination coverage when forming a differential diagnosis of an apparent infectious process. Our research highlights how expanded data about the prevalence of specific infectious disease in refugee populations can allow targeted development of syndromic approaches to diagnosis, providing effective treatment in the absence of high-resource microbial testing. In addition, use of such data would allow targeted deployment of rapid testing resources (for example, for malaria) to refugee centers. Both of these approaches can be rolled out in coordination with community-level services providing care in affected regions and used to assist NGOs and others working with relevant populations. Data can also be used to tailor education campaigns rolled out at the community level in refugee centers.

Lastly, vaccine programs are an important part of practice in conflict regions to mitigate the impacts of displacement and increased exposure to infectious disease. Refugees from conflict are important targets for vaccination efforts aimed at reducing vaccine-preventable disease, but specific interventions have been limited and challenged by the heterogeneity inherent to refugee populations [29]. Working with the network of services which make contact with refugees to provide up to date vaccines to patients in conflict zones could be an impactful strategy.

### Limitations

The retrospective, correlational nature of this study prohibits firm attribution of causality to any predictor. In addition, the number of datapoints for each observed correlation was fairly low, and for this reason multivariate analysis was not pursued to avoid inflating trend strength. Data availability was poor for many disease incidences, and often did not overlap with the most significant portions of conflict data, making it difficult to distinguish spurious correlations from more meaningful associations in some cases.

The UCDP dataset allows spatial resolution of data at very high levels and also permits categorization of the type of fighting involved in each entry. We did not perform detailed analysis of the types of conflicts occurring in target countries. In many cases, fighting may also be cross-border, and involve nations not considered in this study.

Further research could attempt to expand on the fidelity with which spatial relationships are rendered, considering conflict data at the local, rather than national level. The greatest limitation to future examinations of this topic, though, is the limited availability of disease data, and continued strengthening of monitoring programs in the countries considered here will aid efforts to better understand the complex determinants of infectious disease.

## Conclusion

Our findings reinforce existing evidence linking armed conflict to increased incidence of malaria while highlighting more novel associations with tetanus and measles. These results underscore the need for emergency physicians and health systems in both conflict-affected and neighboring regions to prepare for evolving patterns in infectious disease burden. Preparation strategies should include disseminating information about syndromic diagnostic approaches relevant to the presentation of diseases with increased incidence in the specific population addressed, as well as deployment of relevant rapid testing resources to areas where displaced persons may settle or pass through. Physicians can provide specific education to community-level partners targeting diseases which may increase in prevalence following episodes of armed conflict. Efforts to provide vaccination can be aimed at refugees as they move and settle in new areas. . In addition, the observed rise in tetanus cases lends empirical support to the WHO’s Controlled Temperature Chain (CTC) roadmap, emphasizing the urgency of further high-quality research into vaccine safety and efficacy amid disruptions to cold chain infrastructure.

## Dissemination of results

The results of this study will be shared with the creators of the public datasets analyzed for further dissemination.

## CRediT authorship contribution statement

**Andrew Holzman:** Conceptualization, Formal analysis, Writing – original draft. **Daniel Olinga:** Supervision, Writing – review & editing. **Jacob Busingye:** Supervision, Writing – review & editing. **Douglas Rappaport:** Supervision, Writing – review & editing.

## Declaration of competing interest

The authors declared no conflicts of interest.

## References

[1] Arcos González P., Cabria Fernández J., Gan R.K. (2024). Fernández Camporro Á, Cernuda Martínez JA. The epidemiological profile of incidence and mortality from epidemics in complex humanitarian emergencies from 1990 to 2022 - A scoping review. Trop Med Int Health.

[2] Yu Q., Qu Y., Zhang L., Yao X., Yang J., Chen S., Liu H., Wang Q., Wu M., Tao J., Zhou C., Alage I.L., Liu S. (2024). Spatial spillovers of violent conflict amplify the impacts of climate variability on malaria risk in sub-Saharan Africa. Proc Natl Acad Sci U S A..

[3] Bennett B.W., Marshall B.D., Gjelsvik A., McGarvey S.T., Lurie M.N. (2015). HIV Incidence Prior to, during, and after Violent Conflict in 36 Sub-Saharan African Nations, 1990-2012: an Ecological Study. PLoS One.

[4] Tripodi P., Patel P. (2004). HIV/AIDS, peacekeeping and conflict crises in Africa. Med Confl Surviv.

[5] M'Boussa J., Yokolo D., Pereira B., Ebata-Mongo S. (2002). A flare-up of tuberculosis due to war in Congo Brazzaville. Int J Tuberc Lung Dis.

[6] Gebreyohannes E.A., Wolde H.F., Akalu T.Y., Clements A.C.A., Alene K.A. (2024). Impacts of armed conflicts on tuberculosis burden and treatment outcomes: a systematic review. BMJ Open.

[7] Chepkurui V., Amponsah-Dacosta E., Haddison E.C., Kagina B.M. (2021). Characterization of national immunization programs in the context of public health emergencies: a case study of 13 countries in the WHO Africa region. Front Public Health.

[8] Nazir A., Oduoye M.O., Tunde A.M., Hafsat A., Guta J.G., Akilimali A., Elembwe H., Kitumaini C., Onesime J., Bavurhe R.F. (2023). Measles outbreak in Ethiopia amid COVID-19: an effect of war-induced hampering of vaccination and pandemic. Ann Med Surg.

[9] Pickering A.E., Malherbe P., Nambuba J., Bills C.B., Hynes E.C., Rice B. (2023). Clinical emergency care quality indicators in Africa: a scoping review and data summary. BMJ Open.

[10] Mambo S.B., Nja G.M.E., Bunu U.O., Bizimana G.N., M'yisa Makelele A., Sonia F.D., Jones M.K., Sikakulya F.K. Promoting risk communication and community engagement during Mpox outbreak in fragile conflict zones of Eastern DRC. One Health. 2025 Mar 12;20:101012. doi: 10.1016/j.onehlt.2025.101012. 10.1016/j.onehlt.2025.101012 PMC11951864 40160936

[11] UNHCR (2025). Uganda. https://www.unhcr.org/where-we-work/countries/uganda.

[12] The Guardian (2023). Christmas in Africa’s largest refugee Camp. https://www.theguardian.com/global-development/2023/dec/26/christmas-bidibidi-uganda-africa-refugee-camp.

[13] Sundberg R., Melander E. (2013). Introducing the UCDP georeferenced event dataset. J Peace Res.

[14] Immunization dashboard WHO; 2025. https://immunizationdata.who.int/ [accessed 1 July 2025].

[15] (2025). https://www.who.int/data/gho.

[16] (2013). https://www.api.internal-displacement.org/sites/default/files/publications/documents/201309-af-lra-overview-en.pdf?_gl=1%C3%972ml3g7*_ga*MjEyOTI1MzAxOC4xNzQ5MjgwMDcw*_ga_PKVS5L6N8V*czE3NDkyODAwNjkkbzEkZzAkdDE3NDkyODAwNjkkajYwJGwwJGgw.

[17] UNHCR (2012). Recovering from the lord’s resistance army. https://www.unhcr.org/us/news/stories/recovering-lords-resistance-army.

[18] BBC. Rwanda genocide: 100 days of slaughter BBC; 2019. https://www.bbc.com/news/world-africa-26875506 [accessed 1 July 2025].

[19] Jazeera A.l. (2024). https://www.aljazeera.com/news/2024/2/21/a-guide-to-the-decades-long-conflict-in-dr-congo.

[20] Schwikowski M. The long road to reconciliation in Burundi. https://www.dw.com/en/the-long-road-to-reconciliation-after-burundis-civil-war/a-67174207; 2023 [accessed 1 July 2025].

[21] Schwartz S. (2019). Home, again: refugee return and post-conflict violence in burundi. Int Secur.

[22] (2024). Conflict In The Central African republic.

[23] CFR. Instability In South Sudan, https://www.cfr.org/global-conflict-tracker/conflict/civil-war-south-sudan.

[24] (2025). https://www.malteser-international.org/en/our-work/africa/dr-congo/improved-living-conditions-for-south-sudanese-refugees.html.

[25] Dawisha E. (2025). https://www.thinkglobalhealth.org/article/preserving-refugee-health-uganda.

[26] WHO.Beyond the standard cold chain; 2025. https://www.who.int/teams/immunization-vaccines-and-biologicals/essential-programme-on-immunization/supply-chain/controlled-temperature-chain-(ctc [accessed 1 July 2025].

[27] Juan-Giner A., Domicent C., Langendorf C., Roper M.H., Baoundoh P., Fermon F., Gakima P., Zipursky S., Tamadji M., Grais R.F. (2014). A cluster randomized non-inferiority field trial on the immunogenicity and safety of tetanus toxoid vaccine kept in controlled temperature chain compared to cold chain. Vaccine.

[28] Seaman C.P., Kahn A.L., Kristensen D., Steinglass R., Spasenoska D., Scott N., Morgan C. (2022). Controlled temperature chain for vaccination in low- and middle-income countries: a realist evidence synthesis. Bull World Health Organ.

[29] Charania N.A., Gaze N., Kung J.Y., Brooks S. (2020). Interventions to reduce the burden of vaccine-preventable diseases among migrants and refugees worldwide: a scoping review of published literature, 2006-2018. Vaccine.

